# Morphological and molecular characterization of the human breast epithelial cell line M13SV1 and its tumorigenic derivatives M13SV1-R2-2 and M13SV1-R2-N1

**DOI:** 10.1186/s12935-015-0262-5

**Published:** 2015-11-25

**Authors:** Sandra Stempin, Anika Engel, Nora Winkler, Thorsten Buhrke, Alfonso Lampen

**Affiliations:** Department of Food Safety, Federal Institute for Risk Assessment, Max-Dohrn-Str. 8-10, 10589 Berlin, Germany

**Keywords:** Human breast epithelial cell line, M13SV1, Transcriptomics, 3D cell culture, Cellular differentiation, In vitro model

## Abstract

**Background:**

The estrogen receptor-positive M13SV1 breast epithelial cell line was proposed to be a suitable in vitro model for breast cancer research since two derivatives with graduated tumorigenicity—M13SV1-R2-2 and M13SV1-R2-N1—are available for this cell line. In the present study, these three cell lines were comparatively examined for their morphological and their biochemical properties on the molecular level.

**Methods:**

A transcriptomic approach (gene array analysis) was chosen to unravel differences in gene expression among the three cell lines. Network analysis was conducted to identify deregulated signaling pathways. Cellular viability was determined by impedance measurements as well as by neutral red uptake assay. Apoptosis was determined by using a caspase assay. For morphological characterization, cells were grown in three-dimensional cell culture, and cellular differentiation and spheroid formation was followed by immunofluorescence staining by using confocal laser scanning microscopy.

**Results:**

The gene array results indicated that there were only marginal differences in gene expression among the three cell lines. Network analysis predicted the R2-N1 derivative (1) to display enhanced apoptosis and (2) to have a higher migration capability compared to its parent cell line M13SV1. Enhanced apoptosis was confirmed by elevated caspase activity, and increased migration was observed in 3D culture when cells migrated out of the globular spheroids. In 3D cell culture, all three cell lines similarly formed spheroids within three days, but there was no acini formation until day 21 which is indicated by a growth arrest around day 15, cellular polarization, and the formation of hollow lumen inside the spheroids. These characteristics, however, are crucial to study, e.g., the differentiation process of breast epithelial cells in vitro.

**Conclusion:**

Due to the molecular and morphological features, the M13SV1 cell line and its tumorigenic derivatives seem to be less suitable as in vitro models than other cell lines such as the MCF-10A cell line which displays proper acini formation in 3D culture.

**Electronic supplementary material:**

The online version of this article (doi:10.1186/s12935-015-0262-5) contains supplementary material, which is available to authorized users.

## Background

Nowadays, breast cancer research largely depends on the availability of model systems suitable to monitor breast epithelial cell differentiation and crucial characteristics of mammary gland development in vitro. There are two major types of epithelial cells in mammary gland, namely luminal cells and basal or myoepithelial cells. These two cell types originate from the same stem cell population that is also found in the mammary epithelium [1]. For in vitro studies, numerous cell lines have been derived from mammary gland tissue in order to generate permanent cell culture models e.g., for breast cancer research [2]. Among other model systems, a protocol has been reported for the isolation of two types of human breast epithelial cells (HBEC) [3]. Type I HBEC are luminal epithelial cells with stem cell characteristics capable of e.g., differentiating into type II HBEC which have a basal epithelial cell phenotype. The M13SV1 cell line was derived from SV40-immortalized type I HBEC cells [3]. This cell line was reported to retain their stem cell characteristics and, moreover, to be positive in estrogen receptor expression [4]. On top of this, two derivatives of M13SV1 have been generated which display a graduated level of tumorigenicity. The weakly tumorigenic cell line M13SV1-R2-2 was derived from the non-tumorigenic parent cell line M13SV1 by X-ray irradiation. Subsequently, this derivative was transduced with the rat Erbb2 oncogene yielding the highly tumorigenic M13SV1-R2-N1 cell line. Tumorigenicity was evaluated according to the degree of tumor development (number and weight of tumors) in mice that had been subcutaneously inoculated with cells of these three cell lines [5]. Due to all these features, the availability of three estrogen receptor-positive type I HBEC-derived cell lines related to each other with a different degree of tumorigenicity was supposed to be a promising model system to be used for in vitro studies in particular with respect to estrogenic effects in breast cancer development. To further characterize this in vitro model, this study aimed on the elucidation of differences between these three cell lines on the molecular level. For this purpose, a transcriptomic approach was chosen to examine alterations in gene expression among the three cell lines under in vitro conditions in order to unravel deregulated metabolic and/or signaling pathways which may be correlated with the different degree of tumorigenicity in vivo. Moreover, the morphological characteristics of the three cell lines grown in 3D culture were compared with each other.

## Results

A transcriptomic approach was chosen to analyze the differences between the M13SV1 cell line and its tumorigenic derivatives on the molecular level. Total RNA was prepared from the three different cell lines and subjected to gene array analysis. By using the standard settings for gene array data evaluation (fold change ± 2, p < 0.05), it turned out that only a small number of the 24,266 different transcripts represented by the gene array were differentially expressed among the three cell lines (Table 1). As an example, only 40 transcripts were differentially expressed in the weakly tumorigenic R2-2 cell line compared to its non-tumorigenic parent cell line M13SV1. The deregulation of these transcripts, however, was very significant, as still 39 of these 40 transcripts were significantly deregulated when a p-value of 0.001 was applied in the analysis (Table 1). Thus, we decided also to allow more subtle deregulations and therefore to use a fold change of 1.5, but in combination with a stringent p-value of 0.001. The use of these settings increased the number of deregulated genes by two- to threefold in comparison to the standard settings (Table 1) resulting in a broader data set to be used in the subsequent network analysis. The lists of deregulated genes (fold change ≥ 1.5; p < 0.001) can be found in the Additional file 1: Tables S1, Additional file 2: Table S2.

**Table 1:** Number of deregulated genes of the gene array experiment

Fold change	p-value	R2-2 vs. M13SV1	R2-N1 vs. M13SV1
2	0.05	40 (15/25)	110 (71/39)
2	0.01	40 (15/25)	109 (71/38)
2	0.001	39 (14/25)	93 (59/34)
1.5	0.05	154 (65/89)	418 (290/128)
1.5	0.01	152 (65/87)	372 (250/122)
1.5	0.001	122 (50/72)	217 (126/91)

Different settings according to fold change and p-value are compared. The values in the brackets indicate the number of upreglulated genes followed by the number of downregulated genes

The software package Ingenuity Pathway Analysis (IPA) was employed to gain insight into molecular metabolic and signaling pathways that are differentially regulated among the three cell lines. The IPA software is a literature-based database that allows a comparison of one’s own experimental data with published data. In our analysis, this comparison was restricted to published experimental data obtained only with human breast cancer cell lines or with primary isolates from human mammary gland. It turned out that although 122 gene transcripts were significantly deregulated in the R2-2 cell line compared to its parent cell line M13SV1 (Table 1) there were no metabolic or signaling pathway or any biological function significantly affected according to the IPA analysis. Thus, on the molecular level these two cell lines seem to be very similar to each other. In the case of the highly tumorigenic R2-N1 cell line, the deregulation of 217 gene transcripts compared to the M13SV1 parent cell line had an impact on a few signaling pathways which in turn affected a number of biological functions. IPA analysis revealed an activation of ERBB2 signaling as well as an activation of the TGFB1- and RAF1-dependent pathways, whereas WISP2 signaling was inhibited in R2-N1 cells compared to the parent cell line. Based on these findings, the IPA software predicted a decreased cellular viability corroborated by the prediction of an increased cell death and of elevated apoptosis in R2-N1 vs. M13SV1. Moreover, the software predicted an enhanced capability to migrate for the highly tumorigenic derivative R2-N1 compared to its parent cell line. The results of this analysis which are summarized in Table 2 are statistically significant (p < 0.05), however, according to the z-scores which in no case reached the threshold of ±2 it has to be stated that the predicted effects are based only on weak statistical power.

**Table 2:** Summary of the network analysis

	Activation status	p-value	z-score
Upstream regulator			
ERBB2	Activated	1.27E−05	1.117
RAF1	Activated	7.60E−07	0.447
TGFB1	Activated	1.22E−03	0.681
WISP2	Inhibited	3.29E−03	−0.762
Biological function			
Migration of breast cancer cell lines	Activated	2.44E−04	1.313
Cell viability of breast cancer cell lines	Inhibited	1.64E−02	−1.346
Cell death of breast cancer cell lines	Activated	3.52E−02	1.339
Apoptosis of breast cancer cell lines	Activated	2.87E−02	1.116

Upstream regulators and biological functions that are activated or inhibited in the R2-N1 cell line in comparison to the parent cell line M13SV1 according to the results of the IPA analysis

**Fig. 1:**
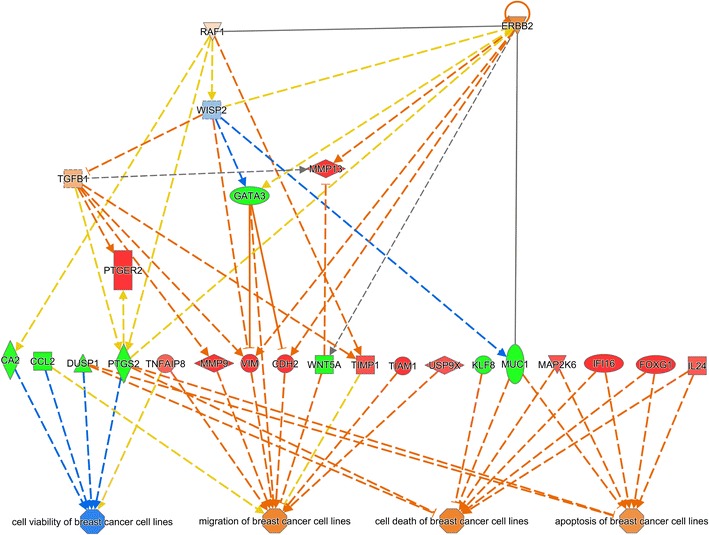
Network generated by the IPA software on the basis of the gene array data. The following settings were used for IPA analysis: species = human; organ = mammary gland or breast cancer cell line; only experimental data were allowed. Genes that were upregulated in R2-N1 cells compared to M13SV1 cells (fold change ≥ 1.5; p < 0.001) are highlighted in *red*, and downregulated genes are highlighted in *green*. *Blue colour* indicates that the IPA software predicted the respective gene to be downregulated whereas *orange-coloured* genes were predicted to be upregulated. The gene names are given, and the function of the respective gene products are indicated by the shape of the *symbols*: enzyme (*vertical diamond*); transcription factor (*horizontal oval*); cytokine (*square*); peptidase (*horizontal diamond*); growth factor (*dotted square*); phosphatase (*triangle up*); kinase (*triangle down*); other (*circle*). The relationship between two genes are indicated by a *solid arrow* (direct effect on gene expression), by a *dotted arrow* (indirect effect), or by a *solid line* (direct protein–protein interaction). In the case of the *arrows,* *orange colour* indicates that the IPA software predicts an upregulation of the downstream gene or an activation of the downstream function whereas *blue colour* indicates the prediction of a downregulation or inactivation, respectively. *Yellow-coloured arrows* indicate inconsistency between the prediction made by IPA based on the experimental data and the literature data

By using the IPA software a molecular network was built that links the relevant deregulated genes to the upstream regulators as well as to the downstream biological functions (Fig. 1). According to this network, activation of ERBB2 and TGFB1 leads to a deregulation of a number of genes (MMP9, VIM, CDH2, WNT5A) which in turn results in an enhanced migration capability of the R2-N1 cell line compared to the parent cell line M13SV1. The predicted decreased cellular viability as well as increased cell death and apoptosis of this cell line are commonly due to the deregulation of a number of additional genes (DUSP1, PTGS2, MUC1, MAP2K6, IFI16, FOXG1, IL24) which are linked to the upstream regulators to a minor degree.

**Fig. 2:**
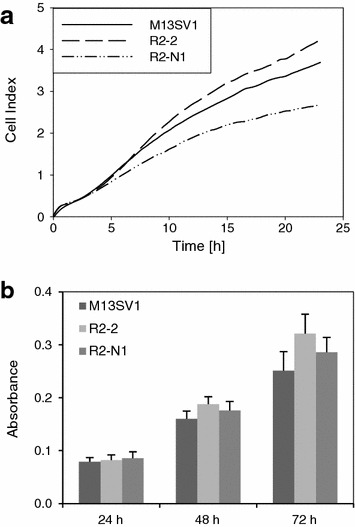
Cellular viability of the three cell lines M13SV1, R2-2, and R2-N1. **a** Growth curves of the three cell lines as obtained by continuous impedance measurement by using the xCELLigence system (Roche); **b** Neutral red cytotoxicity assay. Cells of the three cell lines were seeded in 96-well plates (20,000 cells per well), and the neutral red assay was conducted after 24, 48, and 72 h, respectively. The values are the mean of three independent experiments (+SD)

**Fig. 3:**
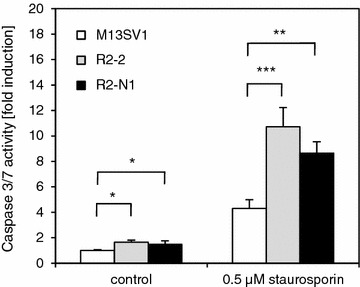
Apoptosis as determined with a caspase 3/7 activity assay. Cells of the three cell lines were seeded in 96-well plates (15,000 cells per well). Cells were treated with 0.5 µM staurosporin to induce apoptosis (*right bars*). Caspase 3/7 activities were determined by using the Caspase-Glo 3/7 Assay (Promega). The values are the mean of three independent experiments (+SD) and were normalized against the value of the untreated M13SV1 cells that was set to 1. Student’s *t*-test; *p < 0.05; **p < 0.01; ***p < 0.001

Growth characteristics were examined and apoptosis assays were conducted in order to experimentally prove the predictions made by the IPA software with respect to the biological functions cell viability, cell death, and apoptosis. Growth curves obtained by continuous impedance measurement revealed that R2-N1 cells seemed to grow slightly slower than the two other cell lines (Fig. 2a), possibly pointing to a decreased cellular viability. The neutral red cytotoxicity assay, on the other hand, indicated that there were no significant differences in the viability between the three cell lines (Fig. 2b). Finally, simple trypan blue staining and counting of living and dead cells revealed that there were approximately 90–95 % living cells in growing cultures of the three cell lines, and there was no indication for any difference in cellular viability (data not shown). Apoptosis was examined by determining caspase activities in the three cell lines. Indeed, the derivatives R2-2 and R2-N1 displayed higher basal caspase activities compared to the parent cell line M13SV1 (Fig. 3). Moreover, induction of apoptosis by staurosporin was significantly more pronounced in the two derivatives than in the parent cell line. These findings are in line with the predictions made by the IPA software.

To further characterize the molecular differences between the three cell lines in particular with respect to the increasing tumorigenic potential, qPCR experiments were conducted with a focus on genes related to breast cancer. For this purpose, the Human Breast Cancer & Estrogen Receptor Signaling RT^2^ PCR Array (SABiosciences) was employed to examine the expression of 84 different breast cancer-related genes in parallel. The results of this analysis are summarized in Table 3. Again, it turned out that there are only marginal differences in gene expression between the derivatives R2-2 and R2-N1, respectively, and their parent cell line M13SV1. In the case of the weakly tumorigenic cell line R2-2, only five genes were significantly deregulated compared to M13SV1 cells; these were CCNA1, KIT, KRT19, SERPINA3, and TFF1 (standard settings, fold change ± 2, p < 0.05; see Table 3). When comparing the highly tumorigenic derivative R2-N1 with its parent cell line M13SV1, only three genes (CCNE1, COL6A1, and THBS2) were significantly deregulated (Table 3). These genes, however, were not significantly affected in the transcriptomic approach. According to the gene array data, none of these genes was deregulated by a factor of at least 1.5-fold (data not shown). On the other hand, the PCR array data were suitable to confirm the results of the gene array experiment for some of the genes depicted in Fig. 1. Four of these genes, ERBB2, GATA3, PTGS2, and MUC1, were also represented by the PCR array, and in all four cases the direction and the level of deregulation between R2-N1 and M13SV1 were comparable between the gene array and the PCR array. All in all, the gene expression data obtained with the whole genome array as well as with the breast cancer PCR array lead to the conclusion, that there are only marginal differences between the three cell lines at least at the level of gene expression.

**Table 3:** Results of the Human Breast Cancer & Estrogen Receptor Signaling RT^2^ PCR Array experiment

Gene name	Gene symbol	R2-2 vs. M13SV1		R2-N1 vs. M13SV1	
		Fold regulation	p-value	Fold regulation	p-value
Androgen receptor	AR	4.59	0.096	4.48	0.230
BCL2-associated agonist of cell death	BAD	−1.20	0.723	1.26	0.675
BCL2-associated athanogene	BAG1	−1.18	0.545	−1.39	0.043
B-cell CLL/lymphoma 2	BCL2	−1.62	0.197	−2.65	0.088
BCL2-like 2	BCL2L2	1.02	0.914	1.94	0.246
Complement component 3	C3	1.17	0.733	1.14	0.890
Cyclin A1	CCNA1	−2.32	0.009	1.72	0.010
Cyclin A2	CCNA2	1.53	0.069	1.25	0.489
Cyclin D1	CCND1	1.26	0.879	1.13	0.836
Cyclin E1	CCNE1	1.69	0.078	2.11	0.038
CD44 molecule (Indian blood group)	CD44	1.20	0.740	1.30	0.572
Cadherin 1, type 1, E-cadherin (epithelial)	CDH1	1.29	0.493	1.29	0.537
Cyclin-dependent kinase inhibitor 1A (p21, Cip1)	CDKN1A	−1.84	0.004	1.39	0.190
Cyclin-dependent kinase inhibitor 1B (p27, Kip1)	CDKN1B	1.52	0.286	−1.08	0.764
Cyclin-dependent kinase inhibitor 2A (melanoma, p16, inhibits CDK4)	CDKN2A	−1.15	0.056	−1.08	0.998
Claudin 7	CLDN7	1.29	0.671	−1.19	0.713
Clusterin	CLU	2.24	0.109	−1.41	0.336
Collagen, type VI, alpha 1	COL6A1	1.60	0.346	4.49	0.010
Catenin (cadherin-associated protein), beta 1, 88 kDa	CTNNB1	2.36	0.139	1.94	0.393
Cathepsin B	CTSB	−1.05	0.817	1.46	0.155
Cathepsin D	CTSD	1.59	0.178	1.04	0.876
Cytochrome P450, family 19, subfamily A, polypeptide 1	CYP19A1	1.06	0.836	1.91	0.154
Deleted in liver cancer 1	DLC1	1.37	0.564	−1.48	0.370
Epidermal growth factor receptor	EGFR	1.41	0.421	1.04	0.986
V-erb-b2 erythroblastic leukemia viral oncogene homolog 2, neuro/glioblastoma derived oncogene homolog (avian)	ERBB2	2.03	0.355	8.15	0.062
Estrogen receptor 1	ESR1	1.59	0.217	−1.60	0.256
Estrogen receptor 2 (ER beta)	ESR2	1.69	0.298	2.10	0.166
Fas (TNF receptor superfamily, member 6)	FAS	−1.06	0.681	−1.16	0.499
Fas ligand (TNF superfamily, member 6)	FASLG	1.38	0.301	1.47	0.109
Fibroblast growth factor 1 (acidic)	FGF1	1.14	0.801	2.94	0.084
Fibronectin leucine rich transmembrane protein 1	FLRT1	1.13	0.483	1.83	0.196
FOS-like antigen 1	FOSL1	−1.14	0.546	1.51	0.192
Gamma-aminobutyric acid (GABA) A receptor, pi	GABRP	2.64	0.138	1.27	0.511
GATA binding protein 3	GATA3	−1.23	0.550	−2.70	0.092
GNAS complex locus	GNAS	1.07	0.827	1.23	0.832
Gelsolin	GSN	−1.09	0.904	1.14	0.731
High mobility group box 1	HMGB1	−1.09	0.455	1.18	0.402
Heat shock 27 kDa protein 1	HSPB1	1.53	0.042	1.16	0.533
Inhibitor of DNA binding 2, dominant negative helix-loop-helix protein	ID2	−1.09	0.677	−1.46	0.380
Insulin-like growth factor binding protein 2, 36 kDa	IGFBP2	1.06	0.794	−3.35	0.262
Interleukin 2 receptor, alpha	IL2RA	1.16	0.404	1.48	0.109
Interleukin 6 (interferon, beta 2)	IL6	−1.99	0.114	1.07	0.884
Interleukin 6 receptor	IL6R	1.49	0.006	1.50	0.145
Interleukin 6 signal transducer (gp130, oncostatin M receptor)	IL6ST	1.62	0.266	1.35	0.547
Integrin, alpha 6	ITGA6	−1.07	0.600	1.17	0.505
Integrin, beta 4	ITGB4	1.20	0.812	−1.02	0.824
Jun proto-oncogene	JUN	1.05	0.997	1.02	0.686
V-kit Hardy-Zuckerman 4 feline sarcoma viral oncogene homolog	KIT	4.73	0.044	4.81	0.233
Kruppel-like factor 5 (intestinal)	KLF5	−1.37	0.301	−1.29	0.399
Kallikrein-related peptidase 5	KLK5	−1.80	0.241	−1.41	0.431
Keratin 18	KRT18	1.48	0.026	1.36	0.042
Keratin 19	KRT19	2.30	0.031	1.87	0.146
Mitogen-activated protein kinase kinase 7	MAP2K7	2.40	0.121	1.58	0.515
Antigen identified by monoclonal antibody Ki-67	MKI67	1.48	0.029	1.19	0.362
Metallothionein 3	MT3	−2.40	0.310	−1.48	0.828
Mucin 1, cell surface associated	MUC1	−1.30	0.540	−1.72	0.289
Nuclear transcription factor Y, beta	NFYB	1.26	0.571	1.20	0.746
Nerve growth factor (beta polypeptide)	NGF	1.17	0.501	1.16	0.484
Nerve growth factor receptor	NGFR	1.53	0.633	2.61	0.173
Non-metastatic cells 1, protein (NM23A) expressed in	NME1	1.21	0.345	1.31	0.300
Pregnancy-associated plasma protein A, pappalysin 1	PAPPA	−1.03	0.682	−2.57	0.085
Progesterone receptor	PGR	−1.06	0.684	−4.92	0.163
Plasminogen activator, urokinase	PLAU	1.16	0.672	1.66	0.113
Phosphatase and tensin homolog	PTEN	−1.13	0.797	1.05	0.735
Prostaglandin-endoperoxide synthase 2 (prostaglandin G/H synthase and cyclooxygenase)	PTGS2	−1.90	0.157	−3.64	0.104
Ras-related C3 botulinum toxin substrate 2 (rho family, small GTP binding protein Rac2)	RAC2	1.71	0.039	2.11	0.052
Ribosomal protein L27	RPL27	−1.02	0.767	1.16	0.254
Secretoglobin, family 1D, member 2	SCGB1D2	1.12	0.697	1.29	0.439
Secretoglobin, family 2A, member 1	SCGB2A1	−2.02	0.084	−1.78	0.142
Serpin peptidase inhibitor, clade A (alpha-1 antiproteinase, antitrypsin), member 3	SERPINA3	10.61	0.008	16.47	0.201
Serpin peptidase inhibitor, clade B (ovalbumin), member 5	SERPINB5	−1.25	0.028	−1.12	0.448
Serpin peptidase inhibitor, clade E (nexin, plasminogen activator inhibitor type 1), member 1	SERPINE1	−1.31	0.206	−1.55	0.434
Solute carrier family 7 (amino acid transporter light chain, L system), member 5	SLC7A5	1.52	0.505	-1.08	0.910
Small proline-rich protein 1B	SPRR1B	−1.13	0.703	−1.32	0.363
Stanniocalcin 2	STC2	−1.42	0.038	−1.23	0.562
Trefoil factor 1	TFF1	−9.16	0.005	1.04	0.858
Transforming growth factor, alpha	TGFA	−1.26	0.376	−1.37	0.387
Thrombospondin 1	THBS1	1.01	0.995	−1.49	0.122
Thrombospondin 2	THBS2	−1.09	0.602	5.09	0.008
Tyrosine kinase with immunoglobulin-like and EGF-like domains 1	TIE1	1.08	0.836	2.10	0.153
Tumor necrosis factor, alpha-induced protein 2	TNFAIP2	1.09	0.782	−1.05	0.921
Topoisomerase (DNA) II alpha 170 kDa	TOP2A	1.75	0.048	1.25	0.526
Tumor protein p53	TP53	1.19	0.690	1.14	0.786
Vascular endothelial growth factor A	VEGFA	1.09	0.588	−1.12	0.785

**Fig. 4:**
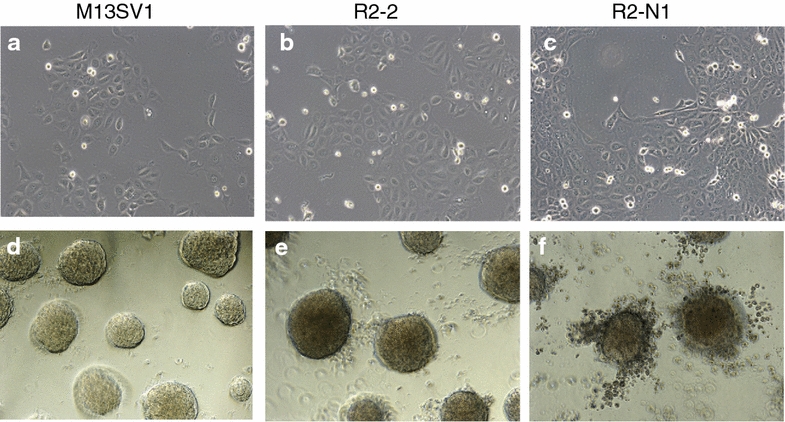
Phase-contrast microscopy. Pictures of the three cell lines as indicated above the *pictures* grown either in 2D culture (**a**–**c**) or in 3D culture (**d**–**f**; day eleven after seeding)

In regard to the prediction that the highly tumorigenic cell line R2-N1 has an enhanced migration capability compared to the parent cell line, the growth characteristics and the morphology of the three cell lines were compared with each other. Under standard cell culture conditions, there were no apparent differences in cell growth and cell morphology when the cells grew in two dimensions on the bottom of cell culture flasks (Fig. 4a–c). In the three-dimensional (3D) cell culture, all three cell lines formed spheroids within three days. These spheroids continuously grew at least until day 21, and there was no growth arrest observed for any of the cell lines (Fig. 4d–f). The only difference among the three cell lines was the observation of R2-N1 cells starting to migrate out of the spheroids around day eleven (Fig. 4f), supporting the prediction that this cell line has an enhanced migration capability compared to the parent cell line. However, migrating cells leaving the spheroids were also observed to a minor degree for the other two cell lines around day 15 (data not shown), indicating that already the parent cell line M13SV1 has a migration capability.

**Fig. 5:**
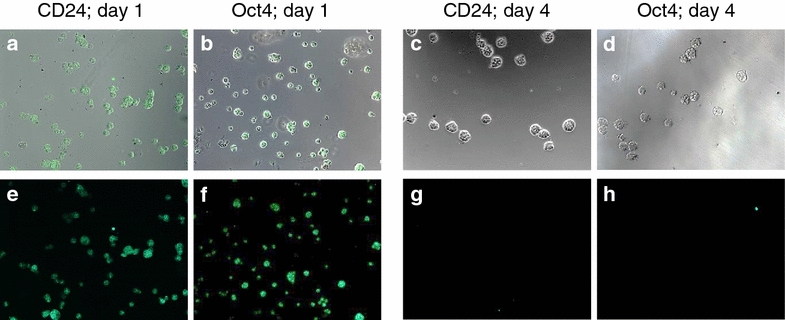
Expression of stem cell markers in M13SV1 cells. Cells were grown in 3D culture and stained either against the stem cell marker CD24 or Oct4, both at day 1 as well at day 4 after seeding as indicated above the *pictures*. **a**–**d** Overlay of the phase-contrast picture and the GFP stain; **e**–**h** GFP stain only

**Fig. 6:**
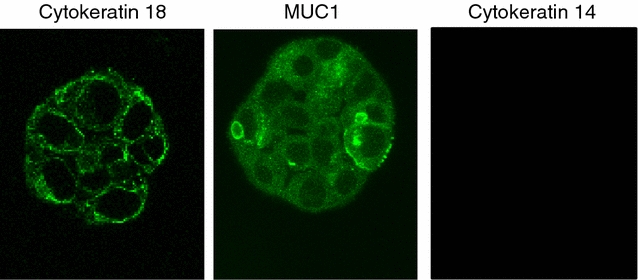
Expression of luminal and basal epithelial cell markers in the M13SV1 cell line. Cells were grown in 3D culture and stained at day 4 after seeding against the luminal markers Cytokeratin 18 or MUC1, or against the basal marker Cytokeratin 14 as indicated above the *pictures*

**Fig. 7:**
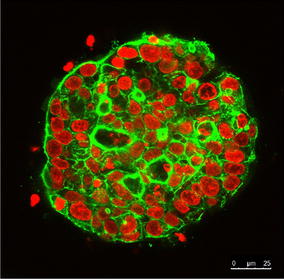
Representative picture of a M13SV1 spheroid grown in 3D culture for 21 days. Nuclei were stained with SYTOX^®^ *Orange* Nucleic Acid Stain, and the cytoskeleton was stained with Alexa Fluor 488 Phalloidin 488. The picture was obtained with a confocal microscope and shows a cut through the middle layer of the spheroid

Confocal microscopy was applied to further characterize the biochemical and morphological features of M13SV1 cells grown in 3D culture. Antibody staining revealed that stem cell marker such as CD24 and Oct4 were present only at day one after seeding, and that these markers were lost around day 4 in the course of spheroid growth (Fig. 5). Luminal markers (Cytokeratin 18, MUC1) were expressed right from the beginning (day one) and were present at least until day 21 whereas expression of basal markers such as Cytokeratin 14 was not detected during this period (Fig. 6). Moreover, the formation of a hollow lumen inside the spheroids due to apoptosis of the inner cells as described for other mammary epithelial cell lines such as MCF-10A [6] was not observed for the M13SV1 cell line. Within 21 days M13SV1 cells formed large spheroids of at least 100 µM in diameter that were completely filled with cells (Fig. 7), and there was no indication for any organized structure within the spheroids that would point to the formation of well-organized acini as described for, e.g., the MCF-10A cell line. Accordingly, antibody staining against active Caspase-3 as a marker for apoptosis was negative (not shown). Finally, staining against specific polarization marker such as Integrin alpha 6 was also negative (not shown), indicating that M13SV1 cells do not differentiate into polarized cells as described, e.g., for MCF-10A cells [6].

## Discussion

The development of well-defined in vitro cell culture models for breast cancer research is a crucial issue not only for fundamental research on mammary gland carcinogenesis but also in terms of, e.g., testing of chemical substances which may have an impact on breast cancer development. With a focus on endocrine disruptors and the examination of potential carcinogenic properties of estrogenic compounds, estrogen-responsive cell lines may be preferred for the development of an in vitro test system. The M13SV1 cell line was regarded as a promising cell culture model as it was shown to be ER positive and even retained some stem cell characteristics. This feature might be regarded as an additional advantage with respect to the stem cell theory which says that numerous tumors originate from stem cells [7–9]. Indeed, M13SV1 cells have already been successfully used for toxicity testing of a number of substances. As an example, it was shown by a transcriptomic approach that treatment of the cells with 2,3,7,8-tetrachlorodibenzo-*p*-dioxin (TCDD) resulted in Akt and ERK2 activation which in turn stimulated cellular proliferation [10]. The authors concluded from their transcriptomic data that the pattern of alteration in gene expression was indicative for TCDD-induced breast tumorigenesis. The two tumorigenic derivatives of M13SV1, however, were not included in this study. In another example, it was shown that treatment of the cells with all-trans retinoic acid resulted in decreased proliferation of M13SV1 cells and of its weakly tumorigenic derivative R2-2, but proliferation was not affected in the highly tumorigenic cell line R2-N1 [11]. Finally, the molecular effects of the carcinogen 7,12-dimethylbenz(*a*)anthracene (DMBA) on the three cell lines were examined, and it was shown that the non-tumorigenic cell line M13SV1 was more susceptible to the mutagen than its tumorigenic derivatives in terms of, e.g., oxidative DNA damage, formation of DNA adducts, and alteration of expression of specific cancer-related genes [12].

The aim of the present study was to compare the M13SV1 cell line with its two tumorigenic derivatives on the molecular level. Thus, the transcriptomes of the three cell lines were compared with each other in order to identify the differences in gene expression which were supposed to be related to the different tumorigenic phenotypes. It has been reported in an earlier transcriptomic study that immortalization of type I HBEC with SV40 DNA to give the M13SV1 cell line was accompanied by the deregulation of 148 genes with a fold change of at least twofold [13], however, a transcriptomic comparison of the M13SV1 cell line with its tumorigenic derivatives has not been reported so far. Although 3D cell culture undoubtedly has a number of tremendous advantages over conventional 2D-cultured cells (see below), we decided to use 2D-cultured cells for the transcriptomic approach for some practical reasons. With respect to a possible use of the cell lines for future high throughput in vitro toxicity testing, 2D culture is much cheaper and much faster than 3D-cultured cells which need approximately 20 days for full differentiation. Moreover, compared to 3D-cultured cells, 2D culture yields much more cell material for subsequent studies. Finally, Kenny and colleagues reported for a number of breast epithelial cell lines that the transcriptomes of 2D-cultures cells are very similar to the transcriptomes of the same cell line cultured under 3D conditions [14]. Our analysis, however, revealed that there are only marginal differences between the gene expression patterns of the three cell lines cultured under 2D conditions. In the case of the weakly tumorigenic cell line R2-2, only 122 out of 24,266 transcripts were significantly deregulated by a factor of at least 1.5-fold compared to its parent cell line M13SV1. The R2-2 cell line was derived from M13SV1 cells by X-ray irradiation, and the exact genetic alterations resulting from this treatment are unknown, however, the tumorigenicity which distinguishes R2-2 cells from M13SV1 cells in vivo is obviously not reflected on the transcriptomic level under in vitro conditions. Thus, post-transcriptional alterations and/or specific interactions with neighboring cells in the in vivo situation may be more relevant for the development of the tumorigenic phenotype. The R2-N1 cell line, on the other hand, was derived from R2-2 cell by a specific genetic modification, namely the introduction of the rat Erbb2 oncogene into the genome. Again, there were surprisingly few transcriptional alterations as only 217 transcripts were significantly deregulated by a factor of at least 1.5-fold between R2-N1 cells and M13SV1 cells. Indeed, the pattern of deregulated genes partly correlated with ERBB2 activation, however, the predicted alterations in biological functions such as increased apoptosis and decreased cellular viability were of only weak statistical power and were hardly reproducible with biological assays. Again, there were only marginal differences in gene expression in vitro between R2-N1 cells and the M13SV1 parent cell line which do not reflect the strong differences with respect to tumorigenicity of these two cell lines under in vivo conditions. Taken together, the transcriptomic approach revealed that there is no obvious set of biomarkers which might have been useful in regard to establish an in vitro test system based on these three cell lines. The use of a PCR-array with specific genes relevant for breast cancer and estrogen receptor signaling did also not unravel strong differences among the three cell lines, again leading to the conclusion that the three cell lines are very similar to each other on the transcriptomic level under 2D conditions.

In order to establish an in vitro test system for human breast epithelial cell lines suitable to monitor substance-induced deregulation of the differentiation process it is crucial to use a cell line which differentiates in 3D culture as similar as possible compared to the in vivo situation in regard to mammary gland formation. Kenny and colleagues have classified 25 breast cell lines into four groups according to their colony morphology [14]. Due to this classification scheme, cell lines that belong to the so-called “round” class seem to be most appropriate to be used for an in vitro test system. Cell lines with a “round” phenotype are characterized by the formation of polarized, growth-arrested colonies with many morphological features of mammary acini such as the formation of a hollow lumen due to the fact that the inner cells of the spheroids undergo apoptosis. Due to these features, cell lines with a “round” phenotype display the most complex differentiation program which is very similar to HBEC differentiation in vivo. The M13SV1 cell line, however, belongs to the so-called “mass” class according to the classification by Kenny et al. [14]. Cells with a “mass” phenotype still form spheroids, but there is no polarization of cells, no growth arrest, and no formation of a hollow lumen. In fact, the M13SV1 cell line as well as its two tumorigenic derivatives belong to the “mass” class and display very similar morphological features when grown in 3D culture. Finally, the presence of distinct stem cell markers was regarded as an advantageous feature of M13SV1 cells, however, these markers are rapidly lost in 3D culture in the course of spheroid growth.

Thus, in conclusion, our analysis revealed that the M13SV1 cell line and its two tumorigenic derivatives seem to be less suitable for the development of an in vitro test system than other permanent HBEC cell lines that belong to the “round” class such as the MCF-12A cell line or the MPE-600 cell line [14].

## Methods

### Cell culture

The HBEC lines M13SV1, M13SV1-R2-2 (designated as R2-2 cells in the following) and M13SV1-R2-N1 (designated as R2-N1 cells in the following) were kindly provided by the working group of James E. Trosko (Department of Pediatrics/Human Development, College of Human Medicine, Michigan State University, East Lansing, USA). The cells were routinely cultured in phenol red- and hormone-free Michigan State University-1 (MSU-1) medium [3] (custom-built; Biochrom, Merck Millipore, Berlin, Germany) supplemented with 5 % charcoal stripped fetal bovine serum (PAA, Pasching, Austria), 10 000 units/ml penicillin and 10 mg/ml streptomycin (PAA, Pasching, Austria). All cells were grown in a humidified atmosphere of 37 °C and 5 % CO_2_.

For all experiments, cell cultures at a confluence of about 80–90 % were washed once with phosphate buffered saline (PBS) and trypsinized (0.05 % trypsin/0.02 % EDTA). Cells were resuspended in culture medium and trypan blue staining was used to determine viability before seeding. Only cells with a viability of at least 90 % were used for experiments.

### Sample preparation and purification of total RNA

The HBEC lines were seeded in separate cell culture flasks (25 cm^2^). Purification of total RNA was performed using the RNeasy Mini Kit according to manufacturer’s recommendations (Qiagen, Hilden, Germany). At a confluence of about 80–90 % the cells were washed twice with ice-cold PBS, directly disrupted in lysis buffer and homogenized using QIAshredder spin columns (Qiagen, Hilden, Germany). An additional on-column DNAse digestion was performed on all samples to remove residual DNA (RNase-Free DNase Set; Qiagen, Hilden, Germany). The concentration of RNA was determined with a NanoDrop ND-1000 spectrophotometer (NanoDrop Technologies, Wilmington, DE, USA) and integrity was assessed by using Agilent RNA 6000 Nano LabChip kit with the Agilent 2100 Bioanalyzer according to manufacturer´s protocol (Agilent Technologies, Palo Alto, CA, USA). All samples for the microarray analysis as well as PCR array experiments should have a RNA Integrity Number (RIN), a measure of degradation, of 8 or higher (RIN; 1 = totally degraded, 10 = intact).

### Gene array analysis

The gene array experiment was conducted by Eurofins Medigenomix GmbH (Ebersberg, Germany) by using Human Transcriptome 2.0 Arrays (HTA 2.0). 100 ng of total RNA was used for labelling and hybridization according to the protocols of Eurofins. Three independent biological replicates were processed for each cell line. Lists of differentially expressed genes between two cell lines were generated using the Transcriptome Analysis Console (TAC) 2.0 software (Affymetrix, Santa Clara, CA, USA). The resulting datasets were further processed by using the software Ingenuity Pathway Analysis (IPA; spring release March 2015; Qiagen, Redwood City, CA, USA) which eliminated all unmapped probe sets and which furthermore only allowed the probe set with the highest fold change for further analysis in the case that a single gene was represented by more than one probe set on the microarray.

### PCR array

The Human Breast Cancer & Estrogen Receptor Signaling RT^2^ PCR Array System (PAHS-005E) containing 84 specific primer sets was used according to the manufacturer´s protocol (SABiosciences, Frederick, MD, USA). 1 μg total RNA was reverse-transcribed to cDNA with the RT^2^ First Strand Kit (SABiosciences, Frederick, MD, USA). The cDNA was combined with RT^2^ SYBR Green/ROX PCR master mix (SABiosciences, Frederick, MD, USA), and 10 µl of the mixture was added to each well of the PCR array (384-well format). The plates were run under conditions recommended by the supplier using the ABI-7900HT instrument (Applied Biosystems, Foster City, CA, USA).

Data analysis was performed on four biological replicates per HBEC line using the ∆∆C_t_ method with an online analysis tool provided by the manufacturer of the PCR array (RT^2^ Profiler PCR Array Data Analysis version 3.5) as described in detail elsewhere [15]. Data were normalized against a set of four endogenous control genes (B2M, HPRT1, GAPDH, and ACTB) present on the array. Resulting ∆Ct values were converted into fold changes of tumorigenic cell lines relative to non-tumorigenic M13SV1 cells using the 2^−∆∆Ct^ method. Genes that achieved a p-value <0.05 were considered as statistically significant for the study.

### Impedance measurement with the xCELLigence system

The xCELLigence real-time cell analyzer (RTCA) system (Roche and ACEA Biosciences) is a label-free and non-invasive assay system based on a continuous measurement of electrical impedance to monitor biological processes of living cells in real time. The impedance is affected by the cells attached on the electrodes and gives information about their biological status, including cell number, viability, morphology and the degree of cell adhesion [16].

Growth of the HBEC lines was continuously monitored for at least 24 h using 96-well plates that contain microelectrodes (E-plate 96) and the RTCA SP instrument (Roche, Rotkreuz, Switzerland). The measurement of the impedance background was performed with 100 µl of cell culture medium per well. After adding the cell suspension the final volume in a single well was 200 μL (10,000 cells/well). Electric impedance was recorded every 30 min.

The impedance is displayed as *Cell Index* (CI), a relative and dimensionless value directly influenced by cell attachment, spreading and cell proliferation.

### Cytotoxicity assays

The neutral red cytotoxicity assay was conducted as described in Buhrke et al. [17]. Trypan blue staining was employed to determine the ratio between living and dead cells by using the EVE Automatic cell counter (NanoEnTek, Seoul, Korea).

### Caspase 3/7 assay

The activity of caspase 3 and 7 was detected using the Caspase-Glo 3/7 Assay (Promega, Mannheim, Germany). The cells were seeded in white walled 96-well plates at a density of 15,000 cells per well and allowed to attach. Twenty-four hours after plating cells were treated in triplicate with 0.5 µM staurosporin and DMSO as solvent control (0.5 %). Caspase-3 and -7 activities were determined after 12 h after staurosporin treatment according to the manufacturer’s protocol. Caspase-Glo 3/7 reagent was added (25 µl/well), gently mixed, and incubated for 30 min. The assay is based on the cleavage of a proluminogenic substrate, containing a DEVD sequence, by the caspases 3 and 7 which generates a luminescent signal produced by luciferase. Luminescence, that is proportional to the amount of caspase activity present, was measured using an Infinite M200 PRO multimode reader (Tecan, Männedorf, Switzerland).

### Three-dimensional (3D) cell culture

Approximately 2000 cells per cm^2^ of M13SV1, R2-2, and R2-N1 cells, respectively, were seeded on a 1–2 mm solidified layer of Growth Factor Reduced Matrigel (BD Bioscience, Heidelberg, Germany) according to the manufacturer’s protocol one modification. The original matrigel was diluted with PBS to a final concentration of 5 mg/ml. The cells were grown in MSU-1 medium (see above). The medium was replaced every 2–3 days.

### Microscopy

Light microscopic analysis of the HBEC cell lines was carried out using the Carl Zeiss inverse Axiovert 135 microscope (Zeiss, Berlin, Germany). A confocal laser scanning microscope (DMI6000, Leica, Mannheim, Germany) was used to visualize the immunofluorescent stains of cells grown in 3D culture. All primary antibodies were purchased from Abcam (Cambridge, United Kingdom): anti-CD24 (ab30350), anti-Cytokeratin 14 (ab51054), anti-Cytokeratin 18 (ab668), anti-MUC1 (ab28081), anti-Oct4 (ab19857), anti-active Caspase-3 (ab32042) and anti-Integrin alpha 6 (ab20142). All secondary antibodies were purchased from Life Technologies (Carlsbad, CA, USA): Alexa Fluor^®^ 488 F(ab’)2 Fragment of Goat Anti-Rabbit IgG (A-11070), Alexa Fluor^®^ 546 F(ab’)2 Fragment of Goat Anti-Rabbit IgG (A-11070), Alexa Fluor^®^ 488 F(ab’)2 fragment of goat anti-mouse IgG (A-11017) and Alexa Fluor^®^ 546 F(ab’)2 fragment of goat anti-mouse IgG (A-11018).

The spheroids grown in 3D culture were fixed with 3.7 % paraformaldehyde (Sigma-Aldrich, Taufkirchen, Germany) for 10 min at room temperature. After three washing steps (for 5 min each) with PBS containing 0.1 % Tween-20 (PBS-T), the spheroids were permeabilized by 0.2 % Triton X-100 for 10 min at room temperature. After another washing cycle, the spheroids were blocked by using 10 % bovine serum albumin for 15 min. Subsequently, the spheroids were incubated with the first antibody (dilution 1:100 in PBS) for 1 h at 37° C. Afterwards, the spheroids were washed again with PBS-T and then incubated with a secondary antibody (dilution 1:200 in PBS) for 20 min at 37° C. Unbound secondary antibody was removed by washing the cells three times for 5 min with PBS-T. To counterstain the nuclei and the cytoskeleton the spheroids were incubated with Alexa Fluor 488 Phalloidin (dilution 1:40 in PBS, Molecular Probes) and SYTOX^®^ Orange Nucleic Acid Stain (dilution 1:5000 in PBS, Molecular Probes) for 30 min.

### Statistics

In the gene array experiment, statistically significant deregulated genes were identified by a one-way ANOVA test (p < 0.05). Evaluation of the gene array data with the IPA software yielded z-scores in addition to p-values with respect to the level of deregulation of molecular networks. The z-score indicates how many standard deviations the sample lies away from the mean. Networks with z-scores <−2 are regarded as significantly inactivated, and z-scores >2 indicate significant activation of the respective network. The statistical data analysis of the PCR array was performed by using the web portal software of SABiosciences. Student’s *t*-test was used, and p-values <0.05 were considered statistically significant. The neutral red cytotoxicity data as well as the caspase 3/7 activity results were evaluated by using Student’s *t*-test, and p < 0.05 was regarded as statistically significant.

## Additional files

10.1186/s12935-015-0262-5 Gene expression analysis—list of deregulated genes R2-2 vs. M13SV1 (fold change > 1.5; p < 0.001).
10.1186/s12935-015-0262-5 Gene expression analysis—list of deregulated genes R2-N1 vs. M13SV1 (fold change > 1.5; p < 0.001).
